# World Field Epidemiology Day 2022: Empowering field epidemiologists to strengthen health systems’ preparedness and response to public health threats

**DOI:** 10.2807/1560-7917.ES.2022.27.35.2200692

**Published:** 2022-09-01

**Authors:** Carmen Varela Santos, Adam Roth

**Affiliations:** 1 European Centre for Disease Prevention and Control (ECDC), Stockholm, Sweden

**Keywords:** field epidemiology, training, health systems, preparedness, response

Raising awareness of the key role of in-service field epidemiology training programmes (FETPs) in strengthening public health capacity to better protect the health of populations and advance global health security is one of the goals of the World Field Epidemiology Day on 7 September. The initiative, started in 2021, is led by the Training Programs in Epidemiology and Public Health Interventions Network, TEPHINET, and builds on contributions from more than 60 institutions worldwide. Policymakers worldwide, including in Europe, are encouraged to put in place mechanisms to empower the field epidemiology workforce as a way to achieve stronger and more resilient health systems, prepared and capable of responding to serious health emergencies, outbreaks, and pandemics. The European Centre for Disease Prevention and Control (ECDC), with *Eurosurveillance* and the ECDC Fellowship Programme, is joining the World Field Epidemiology Day initiative for a second year [1].

It is evident that FETP fellows and graduates have been playing a central role in the response to the challenges of the COVID-19 pandemic. Recently, Hu et al. presented the results of a survey of FETPs which shows how field epidemiologists have contributed to COVID-19 preparedness and response. Through quantitative and qualitative analysis, seven main themes were proposed to describe substantial contributions which included: conducting epidemiological activities, managing logistics and coordination, leading risk communication efforts, providing guidance, supporting surveillance activities, training and developing the workforce, and holding leadership positions [2]. Fellows of the ECDC Fellowship Programme, both in the Field Epidemiology path (EPIET) and the Public Health Microbiology path (EUPHEM) have been active in the response to COVID-19 as employees of public health institutes and laboratories in European Union/European Economic Area (EU/EEA) countries that constitute the training sites of the programme [3]. Some fellows have, mainly in collaboration with ECDC or the World Health Organization (WHO), been involved in multi-country cross-border initiatives, such as seroepidemiology and vaccine effectiveness studies, contact tracing, and the analysis of the impact of public health measures, all contributing to ensure the quality of health epidemiological data for decision-making.

This edition of *Eurosurveillance* features an article about an outbreak of cryptosporidiosis associated with drinking water in north-eastern Italy in August 2019. It demonstrates how interdisciplinary on-the-ground field epidemiology, conducting local outbreak investigations and putting together scientific evidence, continues to form the basis of and to drive well informed decision-making. The outbreak investigations highlight the need to implement EU water sanitary legislative tools and perform rapid interventions, and show the value of empowered field epidemiologists working at the forefront of One-Health, at the human, animal and environmental health interface. Based on the findings, there is also a call to strengthen the national health surveillance system for early detection [4].

Promoting and further developing the professional capacities of the public health workforce supports health systems strengthening; thus, training programmes and national strategies for recruiting and maintaining the workforce are critical. Some European countries have established registries to monitor the dynamics of recruitment and retention [5]. Training curricula based on competency frameworks and job profiles that respond to the needs of the health system are essential tools for the empowerment of the field epidemiologists and will help advancing knowledge to strengthen public health policies and interventions. In April 2022, ECDC published an updated list of core competencies in applied infectious disease epidemiology for mid-career applied epidemiologists, grouped into six technical areas that should contribute to field epidemiologists’ professional development and workforce planning in the countries (Box).

Box **Technical areas for core competencies in applied infectious disease epidemiology for mid-career applied epidemiologists**

• Essential methods for applied infectious diseases epidemiology;

• Preparedness, surveillance and response to infectious disease outbreaks;

• Communication and advocacy;

• Practice of infectious disease epidemiology;

• Contextual influences on infectious disease management; and

• Leadership and management.

Source [9].

Qualified field epidemiologists, equipped with adequate skills and competencies, are the best resource to develop the FETPs further and to cascade their knowledge and skills within the workforce and institutions. As advocates and key players in continuous professional development, strong professional and alumni networks such as the EPIET Alumni Network (EAN) and TEPHICONNECT are key to the impact of FETPs. In addition, institutionalised training programmes (i.e. in public health institutes, ministries of health, national and international agencies) with fellows being an integral part of health systems’ surveillance, preparedness and response structures, and incorporating the career path of FETP alumni into the workforce planning, generates high return on investment and enables effective outbreak management and public health emergency response.

The EU has long recognised the importance of a competent field epidemiology workforce that is able to respond to cross-border health threats using agreed methods and standards. In 1995, the EPIET programme was established, followed in 2008 by EUPHEM, constituting the ECDC Fellowship Programme. A main feature of the ECDC Fellowship Programme is its European nature and the fellows’ work at different levels: local when investigating outbreaks in the field; sub-national and national in public health institutes and laboratories; and regional/EU/EEA, fulfilling one of the Fellowship objectives: *to strengthen the surveillance and control of infectious diseases and other cross-border health threats or issues of public health concern in the EU/EEA Member States and at EU level, supporting the implementation of* Decision 1082/2013/EU [6].

Established in 2013–2014 under a contract between ECDC and the European Commission, and today funded by the European Commission’s Directorate-General for Neighbourhood and Enlargement Negotiations, the Mediterranean and Black Sea Programme for Intervention Epidemiology Training (MediPIET) is a core component of the EU Initiative on Health Security. The two-year advanced regional FETP has been co-ordinated by ECDC since September 2021. It aims at strengthening the 21 partner countries’ capacities to respond to health threats and support cross-border cooperation on health security threats through exchange of best practices and lessons learnt. As at September 2022, 32 epidemiologists have already graduated in the programme which involves the training of at least two successive cohorts of fellows to support field epidemiology capacity in the partner countries, with activities such as the assessment of training resources and needs, the strengthening of the MediPIET network and increasing sustainability by creating a training cascade effect in the partner countries.

The FETPs have been on the front lines fighting outbreaks and health emergencies, including COVID-19, and at the same time directly strengthening health systems’ capabilities by developing the field epidemiology workforce skills and competencies [2]. As one of the key standards of human resources capacity under International Health Regulations (IHR), FETPs implemented by public health institutes generate a pool of competent professionals who use applied epidemiology to guide public health interventions. The Roadmap for Field Epidemiology, initiated by an interinstitutional group of partners, marks out the future steps in the FETP development with the vision that public health and global health functions are delivered by an effective and professional workforce through an in-service and mentored approach, leading to stronger and more resilient health systems. Incorporating the lessons learned from the COVID-19 pandemic in curricular updates and plans will be crucial while tackling important challenges like quality assurance, sustainable funding, career paths of graduates, and institutionalising FETPs [7]. Most FETPs started as national programmes, but since health security involves structures and capabilities at all levels, increasing frontline and intermediate programmes, usually organised by sub-national institutions or local public health districts, will be key for empowering field epidemiologists at all levels of the system striving for healthier populations.

The importance of collaboration and empowerment of individuals in multidisciplinary teams justifies training professionals with different backgrounds and complementary expertise and competencies while they serve in preparedness and response activities as one of the essential public health operations [8]. Global threats require a global response, and therefore FETP programmes with a regional and cross-border scope will be an instrument to strengthen health systems preparedness and response capacities to tackle serious health threats and pandemics.

## Note

Carmen Varela Santos is Head of Section Public Health Training at ECDC and member of the Strategic Leadership Group (SLG) for the Global Field Epidemiology Roadmap.

Adam Roth is Head of the ECDC Fellowship Programme.
